# Understanding the transmission of bacterial agents of sapronotic diseases using an ecosystem-based approach: A first spatially realistic metacommunity model

**DOI:** 10.1371/journal.pcbi.1012435

**Published:** 2024-09-10

**Authors:** Ahmadou Sylla, Christine Chevillon, Ramsès Djidjiou-Demasse, Ousmane Seydi, Carlos A. Vargas Campos, Magdalene Dogbe, Kayla M. Fast, Jennifer L. Pechal, Alex Rakestraw, Matthew E. Scott, Michael W. Sandel, Heather Jordan, Mark Eric Benbow, Jean-François Guégan

**Affiliations:** 1 Maladies Infectieuses et Vecteurs: Ecologie, Génétique, Evolution et Contrôle (UMR MIVEGEC), Université de Montpellier (UM), Centre National de la Recherche Scientifique (CNRS), Institut de Recherche pour le Développement (IRD), Institut national de recherche pour l’agriculture, l’alimentation et l’environnement (INRAE), Montpellier, France; 2 Epidémiologie des maladies animales et zoonotiques (UMR EPIA), Université Clermont Auvergne, INRAE, VetAgro Sup, Saint-Genès-Champanelle, France; 3 Epidémiologie des maladies animales et zoonotiques (UMR EPIA), Université de Lyon, INRAE, VetAgro Sup, Marcy l’Etoile, France; 4 Department of Entomology, Michigan State University, East Lansing, Michigan, United States of America; 5 Département Tronc Commun, École Polytechnique de Thiés, Thies, Senegal; 6 Department of Biological Sciences, Mississippi State University, MS, United States of America; 7 Department of Wildlife, Fisheries, and Aquaculture, Mississippi State University, Mississippi, United States of America; 8 Fish and Wildlife Research Center, Mississippi State University, Mississippi, United States of America; 9 Department of Osteopathic Medical Specialties, Michigan State University, East Lansing, Michigan,United States of America; 10 Ecology, Evolution and Behavior Program, Michigan State University, East Lansing, Michigan, United States of America; 11 AgBioResearch, Michigan State University, East Lansing, Michigan, United States of America; Washington State University, UNITED STATES OF AMERICA

## Abstract

Pathogens such as bacteria, fungi and viruses are important components of soil and aquatic communities, where they can benefit from decaying and living organic matter, and may opportunistically infect human and animal hosts. One-third of human infectious diseases is constituted by sapronotic disease agents that are natural inhabitants of soil or aquatic ecosystems. They are capable of existing and reproducing in the environment outside of the host for extended periods of time. However, as ecological research on sapronosis is infrequent and epidemiological models are even rarer, very little information is currently available. Their importance is overlooked in medical and veterinary research, as well as the relationships between free environmental forms and those that are pathogenic. Here, using dynamical models in realistic aquatic metacommunity systems, we analyze sapronosis transmission, using the human pathogen *Mycobacterium ulcerans* that is responsible for Buruli ulcer. We show that the persistence of bacilli in aquatic ecosystems is driven by a seasonal upstream supply, and that the attachment and development of cells to aquatic living forms is essential for such pathogen persistence and population dynamics. Our work constitutes the first set of metacommunity models of sapronotic disease transmission, and is highly flexible for adaptation to other types of sapronosis. The importance of sapronotic agents on animal and human disease burden needs better understanding and new models of sapronosis disease ecology to guide the management and prevention of this important group of pathogens.

## Author summary

Bacterial pathogens responsible for sapronotic diseases represent a threat to public health, accounting for one-third of human infections. However, little is known about variations in infection risk in time and space, hence limiting control strategies development, due to a lack of research on these pathogens. In this study, we developed a first realistic mathematical model to describe the spatial and temporal dynamics of sapronotic bacteria and their interactions with wildlife in aquatic ecosystems. Our model allows identifying the characteristics of wildlife-sapronotic bacteria interactions that play the most important role in determining disease risks in animals and humans.

## 1 Introduction

The SARS-CoV-2 pandemic was an important event marking the beginning of the 21^st^ century [1], a disease with a likely zoonotic origin [2] according to the World Health Organization [3]. Although zoonotic pathogens have been brought to the forefront of scientific and public attention during the COVID-19 pandemic, many other pathogens affecting plant, human, and wild and domestic animals are known to have environmental stages. These range from circulating among animals to freshwater and marine aquatic habitats, as well as soil or plant root systems [4,5,6]. The biodiverse and complex soil and aquatic communities provide a myriad of ecological niches for microorganisms such as bacteria, fungi and viruses. Many soil and freshwater micro-inhabitants are either directly or indirectly involved in ecosystem functions, and some taxa are implicated in regulating organisms that may have detrimental effects [6], including diseases in plants, animals or humans [7,8]. Opportunistic pathogens are important components of soil and aquatic communities, and they may be either transient microbes, present for a part of their life-cycle, or permanent residents within aquatic communities. Some of these pathogens can be directly transmitted when they come into contact with a susceptible host, e.g., the bacterium *Clostridium tetani* causing tetanus in humans, while others require transfer via vectors or host carriers that normally live in soil or water [9]. Other important parameters to be considered include whether pathogen populations in soil or water reach population levels high enough to cause infections, i.e., inoculum potential, and whether they are metabolically able to cause infection in specific or coincidental hosts [10].

Microorganisms associated with primary producers, such as plants, algae, and cyanobacteria, in aquatic and soil communities can exert strong impacts on ecosystem functions because these autotrophs are responsible for a wide range of provisioning, regulating and cultural services [8]. Many may also be important pathogens, such as sapronotic disease agents, which constitute one third of human infectious disease agents [4] including the bacteria causing cholera, anthrax and Buruli ulcer in humans. These microbial forms show a saprophagic existence: growing and reproducing on nutrient matter (e.g., decaying and living organic matter) present in the environment, and sometimes causing important epidemics in host populations [11].

According to [4], the epidemiology of sapronotic diseases differs from other types of infectious diseases, notably zoonotic ones. They are sustained in soil and aquatic habitats where they benefit from decaying and living organic matter, and opportunistically infect human and animal hosts through passive contact or ingestion of cysts, spores or dormant cells. Sapronotic disease agents do not show similar elaborate transmission strategies such as those of contagious pathogens, vector-borne disease agents or trophic chain-transmitted parasites [4]. Overall, their transmission is passive, occasional, and contact with the infected environment generally results in infections in animals or humans without inter-individual transmission, even if some have adapted a host-to-host transitory stage in their life-cycle (e.g., in humans for *Vibrio cholerae*, the causative agent of cholera). Sapronotic agents share some similarities with host-parasite relationships that include a host reservoir that can be sensitive to fluctuating environmental conditions [4]. An understanding of such sapronotic disease agents includes knowledge of the host-parasite-environment dynamic and should consider intra-annual and inter-annual variability. However, as ecological research on sapronoses, particularly those with a life cycle similar to *Mycobacterium ulcerans*, is infrequent and epidemiological models of their transmission are rare, very little information is currently available in the international scientific literature. There is a general need in disease ecology to better understand the ecology and evolution of agents of many diseases, particularly those that are associated with environmental or unknown routes of transmission to humans and other animals [12]. There are several examples of known sapronotic agents, some of which are found within the mycobacteria [9].

Buruli ulcer disease is a neglected skin disease of humans caused by *M*. *ulcerans* and is considered a sapronotic disease [13]. *M*. *ulcerans* is now widely distributed in intertropical and subtropical areas. Infections are prevalent in most intertropical regions, but most are concentrated in sub-Saharan Africa where the majority of observed Buruli ulcer cases occur [14]. *M*. *ulcerans* charges, also known as parasitic load in disease ecology, in central African continental freshwater ecosystems, vary strongly with seasonal periodicity. “Bacterial bloom-like” abundances are observed two to three months after the onset of rainfall periods [15]. The rainy season influences the reproduction of a large diversity of aquatic organisms such as macroinvertebrates and fishes. Free-living *M*. *ulcerans* stages benefit from these favorable environmental conditions anchoring themselves to and reproducing on this myriad of new aquatic organisms, which offer abundant nutrient sources, e.g., especially chitinous components [16]. The bacterial load then decreases gradually with the onset of the dry season, often coinciding with fish mortality and the emergence or aestivation of different macroinvertebrate life stages [17]. Several studies have also demonstrated that variations in *M*. *ulcerans* abundance in space and time depend on aquatic and nearby ecosystem modifications (e.g., deforestation, agricultural development) with disruptions of local species communities resulting in an upsurge of mycobacteria in altered environments [18]. Although both phenological and anthropological events are crucial for a better understanding of how sapronotic agents contribute to disease risk in the realm of modern disease ecology [19], their importance is often overlooked in medical research focused on diagnosis and treatment.

Considered an environmental pathogen, with likely multiple environmental transmission pathways [9], *M*. *ulcerans* serves as an excellent example of how understanding and modeling its ecology can guide the management and prevention of Buruli ulcer disease in humans. Information on the population dynamics of *M*. *ulcerans* has been tracked in Cameroon, central Africa, and Australia [20,21] showing seasonal fluctuations preceding Buruli ulcer cases in human patients [15,21]. Thus, our goal was to develop the first mathematical model incorporating differential equations of species population and community growth, for both host carriers and mycobacteria. This model is based on bacilli cells that, depending on parameter values, are spread through aquatic ecosystems, either as sapronotic agents or as microbiome-residents of their host carrier. Numerous modeling studies on the population dynamics of aquatic species informed our work [22,23,24,25]. However, research on host-parasite population, community dynamics and transmission for sapronotic agents in aquatic systems are still very rare. Here, using dynamical models and appropriately modified models to explore complexities, we analyze sapronotic agent transmission and spread in aquatic ecosystems.

Using a metapopulation model applied to host-disease agent systems and its extension to metacommunities [26], we simulated sapronotic agent transmission, using *M*. *ulcerans* as a relevant illustration. We do this in hydrological systems reproducing the topology of river catchment areas, and taking into account the main components of rivers and associated biodiversity (e.g., riverine segments, tributaries, main river, advection water current, and biodiversity involved in the disease agent life-cycle, as well as migration flows of aquatic species). We modeled host-parasite spatio-temporal dynamics in watersheds but did not include an epidemiological component, i.e., links with human disease cases, which constitutes concurrent work intended for a forethcoming paper. We compared criteria for pathogen persistence and invasion, coexistence with host carriers and population dynamics in aquatic systems, for two types of bacilli life-history strategies, namely saprophytic and microbiome-resident. To our knowledge, this is the first set of metacommunity models of sapronotic disease agent transmission for freshwater ecosystems. We finally argue a need for better understanding animal and human sapronotic agents and their associated diseases, aiming to better predict and prevent sapronoses.

## 2 Materials and methods

### 2.1. Mathematical model

**Fig 1:**
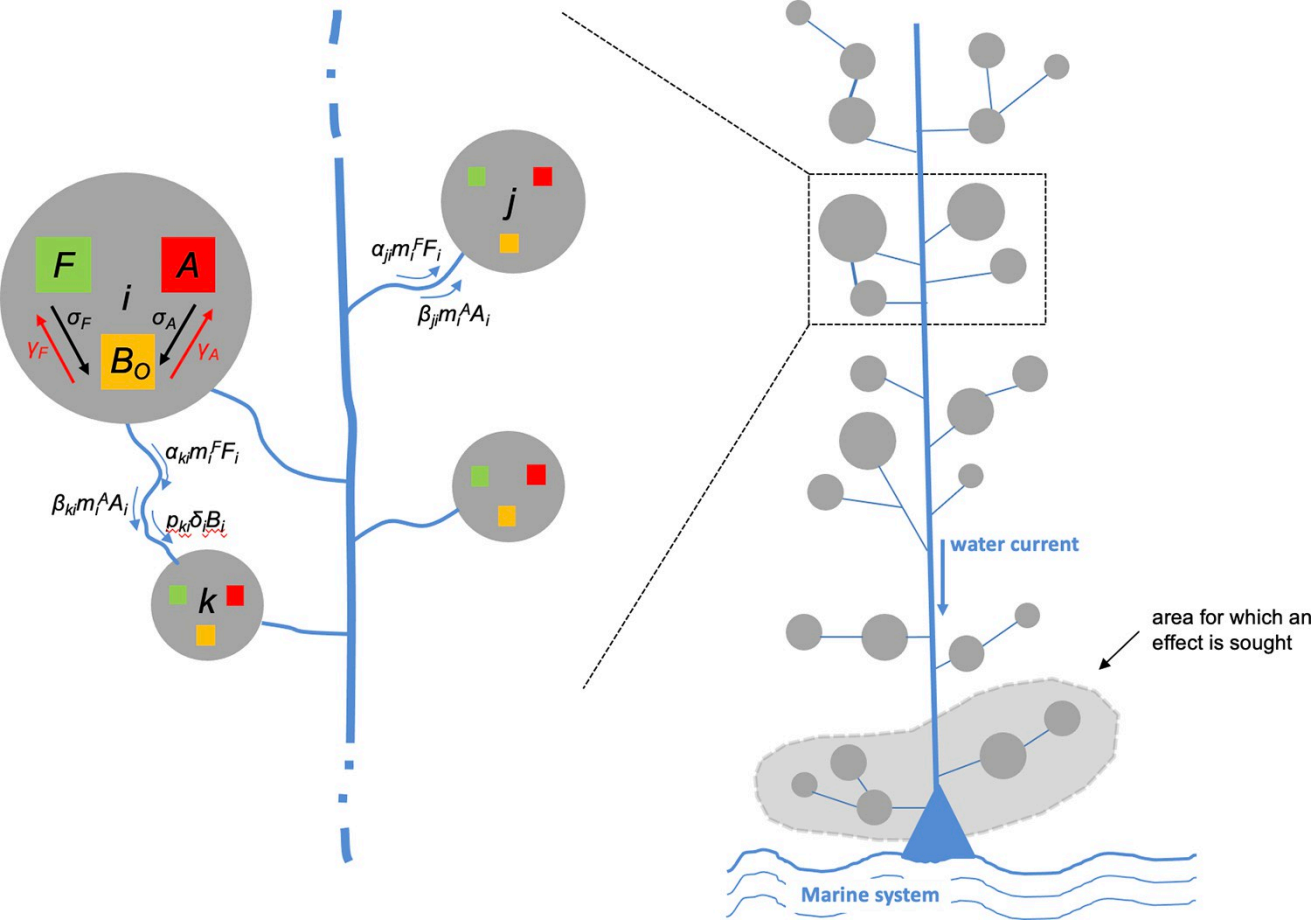
Schematic representation of the metacommunity model. Variables and parameters are defined in Table 1. On the right, a simplistic illustration of the river metacommunity model including 25 different habitat patches represented by grey disks of different sizes, the triangle area corresponds to the river delta and the light grey depicts the downstream area for which an effect is sought. On the left, local population interactions and organism dispersal among habitat patches. The blue lines correspond to the main river and tributaries, and constitutes connectivity in the metacommunity system. ***F***: local freshwater fish subpopulation in patch *i*; ***A***: local aquatic macroinvertebrate subpopulation in patch *i*; ***B***_***o***_: local free-living bacteria subpopulation in patch *i*. The black arrows represent the saprophytic process through decaying organic matter recycling for both fish and aquatic macroinvertebrates. The red arrows describe bacterial adhesion on surface of corresponding living biomass, i.e., fish and macroinvertebrate. The blue arrows show the dispersal of organic biomass and bacteria.

We developed a spatially explicit metacommunity model [27] composed of *n* patches (called subcommunities hereafter), distributed along a river catchment area (Fig 1) and subjected to seasonal fluctuations between rainy and dry seasons. Our spatial model more closely resembles a source-sink metacommunity structure, emphasizing the effect of dispersal of both pathogen propagules and host carriers, than a metapopulation-based model. Source-sink population and community structure results from differences in habitat quality whereas true metapopulation-metacommunity models reflect the patchiness of the environment. Both structures are represented in this work, but the source-sink structure more accurately reflects the population dynamics of free-living bacteria, which occur with the downstream flow of water in river catchment areas, i.e., advection water current. This model is aimed at capturing how the abundance dynamics of free-living bacteria that may accumulate along the nearby coastline, i.e., the alluvial plain, depend on its regional dynamics over the upstream catchment area. We set the number of local patches in the alluvial plain under scrutiny to 5; this constitutes our sink area. For this, we considered upper- and middle-river subcommunities acting as primary and secondary sources for the bacterial persistence and reproduction, i.e., supplying the zones located downstream. In any of these upper river patches, the bacterium is considered to exist in three potential states; as free-living bacteria in the water column or in association with either one of two host carrier types corresponding to freshwater fish or macroinvertebrates [20]. Free-living mycobacterial cells may attach on living organism surfaces, i.e., hereafter called biomass-associated, and can also develop on decaying organic matter and release new free cells, i.e., hereafter called saprophytic stage. We considered that there is no direct transmission through contact between fish-associated and macroinvertebrate-associated mycobacteria. However, pathogen transmission between biomass-associated and free-living stage occurs both ways. Bacterial doubling times differed between free-living forms, fish-associated and macroinvertebrate-associated bacteria. Also, the carrying capacities of biomass-associated bacteria differed between fish and macroinvertebrate compartments (see description of the parameters in Table 1).

**Table 1:** Meaning of the variables and parameters used in the mathematical model.

Notations	Descriptions	Values (unit)
	**State functions**	
*F*_*i*_ *(t)* *A*_*i*_ *(t)* *B*_*F*,*i*_ *(t)* *B*_*A*,*i*_ *(t)* *B*_*o*,*i*_ *(t)*	Concentration of fish carrier biomass in subcommunity *i* Concentration of macroinvertebrate carrier biomass in subcommunity *i* Concentration of fish-associated bacteria in subcommunity *i* Concentration of macroinvertebrate-associated bacteria in *i* Concentration of free-living bacteria in subcommunity *i*	
	**Parameters**	
*n* *r*_*i*_*(t)* ρ_i_(t) *Λ*_i_(t) λ θ ν *η* *μ* m_*i*_^F^ m_*i*_^A^ *δ*_*i*_(t) α_*ij*_ β_*ij*_ p_*ij*_ γ ξ σ_F_ σ_A_ T s_F_ s_A_	Number of subcommunities or local patches Time-dependence fish growth rate for patch *i* Time-dependence macroinvertebrate growth rate for patch *i* Time-dependence free-living bacteria growth rate for patch *i* Fish-associated bacteria growth rate Macroinvertebrate-associated bacteria growth rate Fish mortality rate Macroinvertebrate mortality rate Bacteria mortality rate Fish migration rate for subcommunity *i* Macroinvertebrate migration rate for subcommunity *i* Time-dependent free-living bacteria migration rate for subcommunity *i* Proportion of fish moving from subcommunity *j* to subcommunity *i* Proportion of macroinvertebrate moving from subcommunity *j* to subcommunity *i* Proportion of free-living bacteria moving from subcommunity *j* to subcommunity *i* Bacteria attachment rate on fish biomass Bacteria attachment rate on macroinvertebrate biomass Bacilli release rate from dead fish Bacilli release rate from dead macroinvertebrate Leaching period of free-living bacteria Half saturation Michaelis-Menten constant of the fish-associated substrate Half saturation Michaelis-Menten constant of the macroinvertebrate-associated substrate	25 0.1 (*t*^-1^) 0.45-0.5 (*t*^-1^) 0.05 (*t*^-1^) 0.05 (*t*^-1^) 0.027 (*t*^-1^) 0.1 (*t*^-1^) 0.05 (*t*^-1^) 0.1 (*t*^-1^) 0.27-0.3 (*t*^-1^) 2 2-6 7-10 1 1

**Fig 2:**
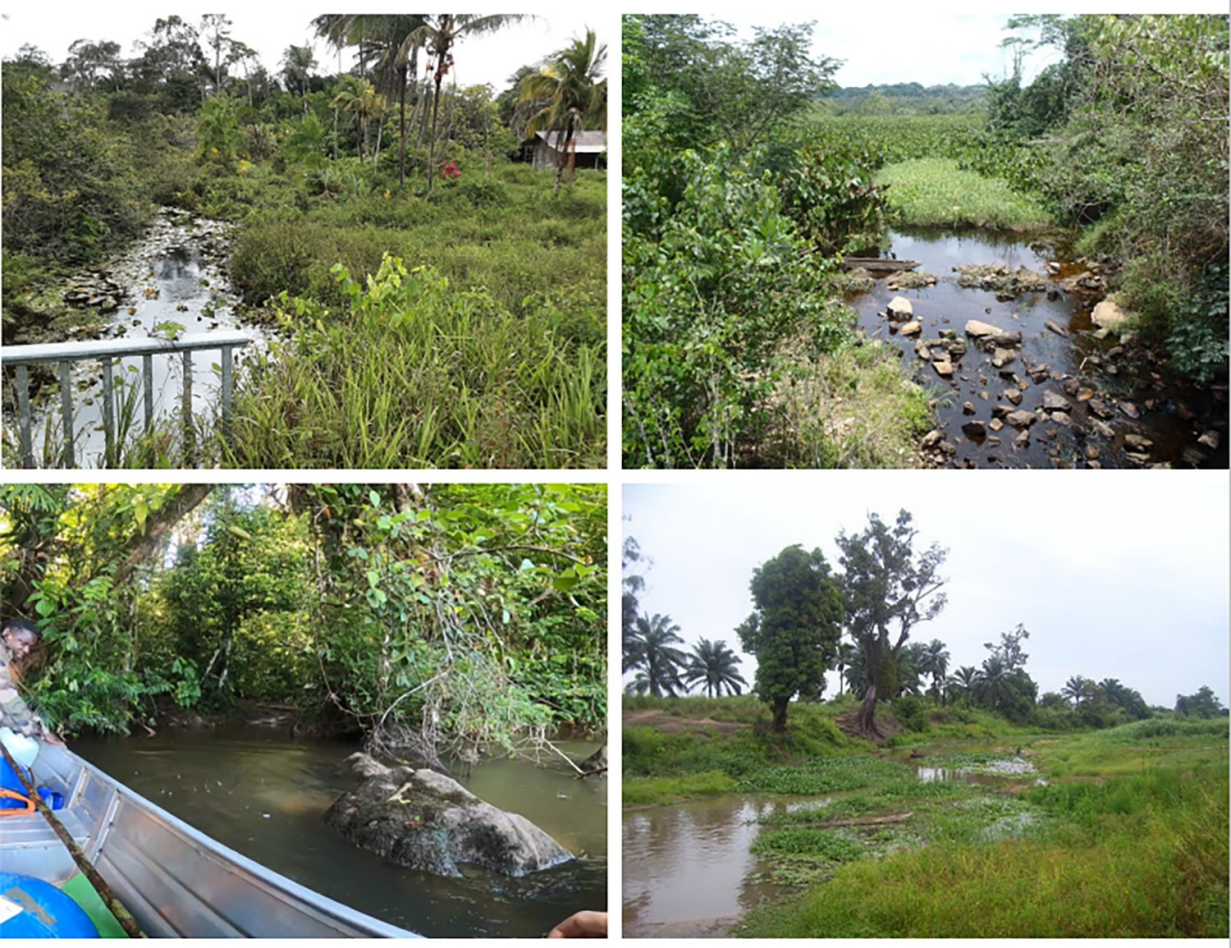
Illustration of different areas favorable for the development of *Mycobacterium ulcerans*, responsible for causing Buruli ulcer, and representative of local patches or subcommunities in the mathematical model developed in this paper. Upper left: lower river area close to the Mana river, French Guiana, South America (photo credit: J-FG, 2021); lower left: upper area of the Sinnamary river, French Guiana (photo credit: MEB, 2021); upper right: middle area of the Nyong river, central Cameroon, Africa (photo credit: J-FG, 2014); lower right: a small stream habitat in Benin, western Africa (photo credit: MEB, 2016). All these sites harbor a diverse community of species, including free-living mycobacteria, freshwater fish and macroinvertebrate species, fish-associated mycobacteria and macroinvertebrate-associated mycobacteria. The different patches from a same catchment area can be more or less connected to each other, depending on geographical distance, according to the schematic illustration in Fig 1.

At time *t*, each patch or subcommunity *i* can either be occupied or unoccupied. We defined a patch as a suitable habitat (see Fig 2) that can sustain a local species community containing free-living stages of bacteria (*Bo*), several fish species with a given local biomass (*F*), several macroinvertebrate species with a given local biomass (*A*), fish-associated bacterial forms (*B*_*F*_) and macroinvertebrate-associated bacterial forms (*B*_*A*_). Movements of fish and macroinvertebrates occur in both directions along the river and tributary segments. By contrast, the dispersal of free-living bacteria is highly seasonal, being null in dry season, and conditioned by the direction of river flow during rainy season. In subcommunity *i*, we denoted by *F*_*i*_ the concentration of freshwater fish species biomass, *A*_*i*_ the concentration of aquatic macroinvertebrate biomass, B_*F*,*i*_ the concentration of fish-associated bacteria, *B*_*A*,*i*_ the concentration of macroinvertebrate-associated bacteria, and *B*_*o*,*i*_ the concentration of free-living bacteria. Population growth is often modeled using the logistic equation proposed by Verhulst [28] because it explicitly takes into account the saturation carrying capacity, i.e., the maximal population size that the environment can support. In the present work, the carrying capacity is instead implicitly incorporated into our mathematical model.

The migration rates of fish and aquatic macroinvertebrates between every two patches *i* and *j* remain constant throughout the year. These migration rates may either be short-distance events (i.e., being non-null among patches located within the same river section) or long-distance events (i.e., being non-null among patches belonging to adjacent river sections). The river flow has no impact on the direction of fish and macroinvertebrate migration events. Biomass-associated bacteria are passively migrating with fish and macroinvertebrate taxa with which they are associated. The range of free-living bacterial dispersion changes with the rainy seasons as follows. With the peak of rainfall during the rainy season, river system reaches its maximum stage height. These conditions promote heavy leaching that washes away everything along the river corridor, resulting hence in the elimination of free-living bacteria from all monitored sites, including upstream secondary sources and alluvial sinks. The end of the rainy season comes with lower river height and slower river flow, hence opening downstream sites where the free-living bacteria from upper areas (issued from new colonization from the primary source and from dead infected biomass) can settle now.

Each subcommunity of species inhabiting a patch presents both its internal dynamics (driven by birth and death events) and external dynamics (driven by dispersal processes). Two seasons per year are considered to adjust for tropical climatic conditions where these mycobacteria are found: one rainy season followed by one dry season. The rainy season corresponds to the onset of the reproductive period for freshwater fish and macroinvertebrates with an aquatic life stage. For the rainy season, the intrinsic demographic fish and macroinvertebrate dynamics within each patch is described by a function that implicitly considers the local patch’s carrying capacity, thus determining a capacity threshold (see below). For the dry season, fish and macroinvertebrate species populations are logically decreasing, with *ν* and *η* representing the respective death rates. Concerning free-living bacteria, they mostly grow in population size during the rainy season according to Garchitorena et al. [20], and biomass-associated bacteria evenly grow all year around, with their carrying capacity implicitly taken into account as a percentage of the host carrier biomass present in a given local patch.

Using the variables and parameters describes in Table 1, we generated the following model, for *i* = 1, · · ·, 25 patches. The seasonal dynamics of fish and macroinvertebrate biomasses are given by:

$$\frac{dF_{i}}{dt}=r_{i}F_{i}-(\nu +m_{i}^{F})F_{i}+\sum_{\begin{array}{c} j=1\\ j\neq i \end{array}}^{n}\alpha_{ij}m_{j}^{F}F_{j}$$ (1)

$$\frac{dA_{i}}{dt}=\rho_{i}A_{i}-(\eta +m_{i}^{A})A_{i}+\sum_{\begin{array}{c} j=1\\ j\neq i \end{array}}^{n}\beta_{ij}m_{j}^{A}A_{j}$$ (2)

The proportion of fish (and macroinvertebrate, respectively) leaving subcommunity **i** at time ***t***, is $m_{i}^{F}F_{i}$ ($m_{i}^{A}A_{i}$, respectively). Now, we define ***α***_***ij***_ and ***β***_***ij***_ as the probability that fish and macroinvertebrate individuals, respectively, migrate into patch **i**, originated from patch **j**. Hence, the flow of fish (and macroinvertebrate, respectively) dispersing from patch **j** to patch **i** is $\alpha_{ij}m_{j}^{F}F_{i}$ ($\beta_{ij}m_{j}^{A}A_{i}$, respectively). By mass conservation, we have the obvious condition ∑_***i***_***α***_***ij***_ = **1** and ∑_***i***_***β***_***ij***_ = **1**.

The seasonal dynamics of fish-associated and macroinvertebrate-associated bacteria are respectively given by:

$$\frac{dB_{F,i}}{dt}=\frac{\lambda F_{i}}{F_{i}+s_{F}}B_{F,i}-(\mu +\nu +m_{i}^{F})B_{F,i}+\frac{\gamma F_{i}B_{o,i}}{F_{i}+s_{F}}+\sum_{\begin{array}{c} j=1\\ j\neq i \end{array}}^{n}\alpha_{ij}m_{j}^{F}B_{F,j}$$ (3)

$$\frac{dB_{A,i}}{dt}=\frac{\theta A_{i}}{F_{i}+s_{A}}B_{A,i}-(\mu +\eta +m_{i}^{A})B_{A,i}+\frac{\xi A_{i}B_{o,i}}{A_{i}+s_{A}}+\sum_{\begin{array}{c} j=1\\ j\neq i \end{array}}^{n}\beta_{ij}m_{j}^{A}B_{A,j}$$ (4)

The intrinsic growth rate of host-associated bacteria depends upon the concentration of the biomass of freshwater fish and aquatic macroinvertebrates serving as substrates for bacterial development. Bacilli have a natural mortality rate *μ*. Here, bacilli attachment to biomass is modelled according to a Michaelis-Menten type functional response, being proportional to both population biomass and free-living bacteria density in the patch considered. We define the maximum success rates for bacilli attachment on fish and macroinvertebrate as *γ* and *ξ*, respectively. The replication rate of biomass-associated bacteria reaches saturation as the surface becomes increasingly populated, but replication continues at a reduced rate. The Michaelis-Menten type functional response is: *λF*_*i*_*/*(*F*_*i*_ + *s*_*F*_), where λ is the maximum production rate of fish-associated bacteria, and *s*_*F*_ is the half-saturation constant. The same functional response was used for macroinvertebrate-associated bacteria reproduction. Moreover, bacilli can reproduce as a saprophytic organism after host death [29,30,31]. Note that biomass mortality also reduces the concentration of biomass-associated bacteria. We suppose, through uniform distribution, that the proportion of bacteria detaching from the host is proportional to the proportion of dead hosts. This corresponds to the term – *νB*_*F*,*i*_ and −*ηB*_*A*,*i*_ in Eqs 3 and 4, respectively.

The seasonal dynamics of free-living bacteria is given by:

$$\left\{ \begin{array}{r} \frac{dB_{o,i}}{dt}=\Lambda_{i}B_{o,i}-(\mu +\delta_{i})B_{o,i}-\frac{\gamma F_{i}B_{o,i}}{F_{i}+s_{F}}-\frac{\xi A_{i}B_{o,i}}{A_{i}+s_{A}}+\sigma_{F}\nu B_{F,i}+\sigma_{A}\eta B_{A,i}+\sum_{\begin{array}{c} j=1\\ j\neq i \end{array}}^{n}p_{ij}\delta_{j}B_{o,j},if\;t\notin T\\ B_{o,i}=0,if\;t\in T \end{array}\right.$$ (5)

The reproduction of free-living bacteria is determined by the growth rate *Λ(t)*. The proportion of free-living bacteria leaving patch ***i*** at time ***t***, is ***δ***_***i***_***(t)***. Conversely, we define ***p***_***ij***_ the probability that bacilli cells migrate into patch ***i***, originated from patch ***j***. Thus, the flow of bacilli dispersing from subcommunity ***j*** to subcommunity ***i*** is ***p***_***ij***_***δ***_***j***_***(t)B***_***o*,*j***_. By mass conservation, one also obtains ∑_***i***_***p***_***ij***_ = **1**. Dead fish and macroinvertebrate bodies are recycled by saprophytic bacteria, and thus death biomass releases new free bacteria in the environment; this leads to the terms ***σ***_***F***_***νB***_***F*,*i***_ and ***σ***_***A***_***ηB***_***A*,*i***_ in Eq 5. The sigma terms (***σ***_***A***_ and ***σ***_F_) reflect overall bacterial growth within the dead host. Here, the parameter T corresponds to the leaching period of free-living bacteria from the river to the sea, without any possibility for these bacteria to settle in the alluvial plain. After the leaching period, as the water level drops, it facilitates the migration and settlement of free-living bacteria from upstream to downstream areas. Towards the end of the rainy season, these free-living bacteria can also colonize intermediate zones.

### 2 2 Simulations parameters

The regional host-bacterial metacommunity consists of a network of 25 different local sub-communities as illustrated on Fig 1. Local species communities form patches that are connected to the overall catchment area metacommunity depending on the ability of species to disperse at short or long distance. In the situation where the patches are relatively homogeneous, the scale of biomass dispersion has limited impacts on the abundance of bacteria downstream. Here we assume that freshwater fish can disperse over medium and long distances (i.e., among adjacent sections of the river) and aquatic macroinvertebrates can disperse locally (i.e., within river sections). Conditions for aquatic macroinvertebrate dispersing regionally roughly yielded similar results and for brevity were not illustrated here. The initial conditions are taken as follows:

$$F_{i}(0)=0.05,\;A_{i}(0)=0.05,\;for\;i=1,\ldots ,25;$$

and the system begins with only a single upstream bacterial source, i.e.,

$$U_{1}(0)=0.02,\;and\;U_{i}(0)=0,\;for\;i=2,\ldots ,25.$$

**Fig 3:**
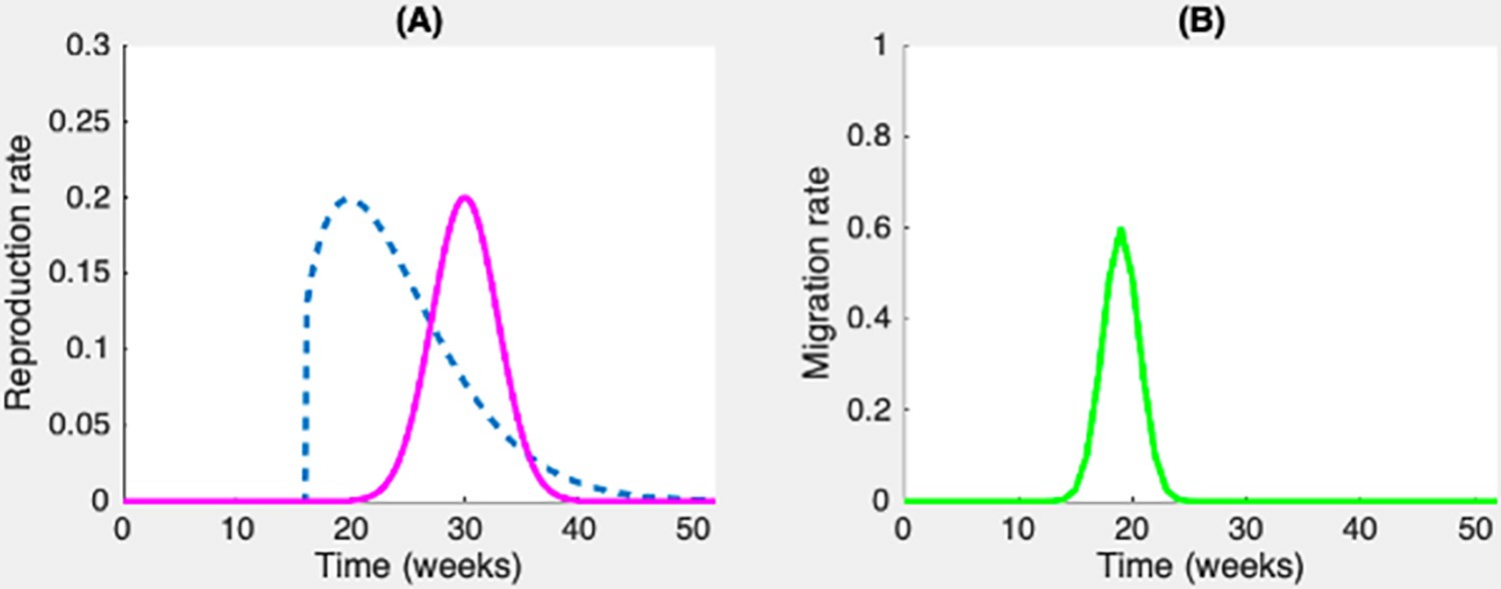
Time-dependence of biomass and free-living bacteria reproduction rates, and of free-living bacteria migration rate. (A) The blue dashed line represents the overall biomass reproduction rate, while the purple line represents the free-living bacteria reproduction rate. (B) The green line represents the free-living bacteria migration rate. Note that the *y* scales are different for the two subfigures.

Fig 3 shows the seasonal variations in the reproduction of biomass for host carriers and free-living bacteria (panel A) and in the migration of free-living bacteria (panel B). We have performed simulations over 9 years using Matlab software (ver. 8.6). The seasonal and decadal dynamics of rainfall, water volume in the catchment area and mycobacteria are consistent with, and mirror, the observations produced by Garchitorena et al. [15,20] and Landier et al. [32]. In our modeling, each year starts with a single rainy season period (30-week long), followed by one dry season period (22-week long). The leaching period of free-living bacteria T from the river to the sea occurs each year at weeks 7–10.

## 3 Results

The mathematical model allowed i) an analysis of how changes in some parameters (i.e., *ξ***, *θ*** and ***σ***_A_) impact the abundance of free-living bacteria in the lower alluvial plain area (i.e., sensitivity analyses) and ii) a quantitative assessment of organic biomass (i.e., fish and macroinvertebrate) influence on the alluvial plain area abundance of free-living bacteria.

### 3.1. Bacterial sources effects

**Fig 4:**
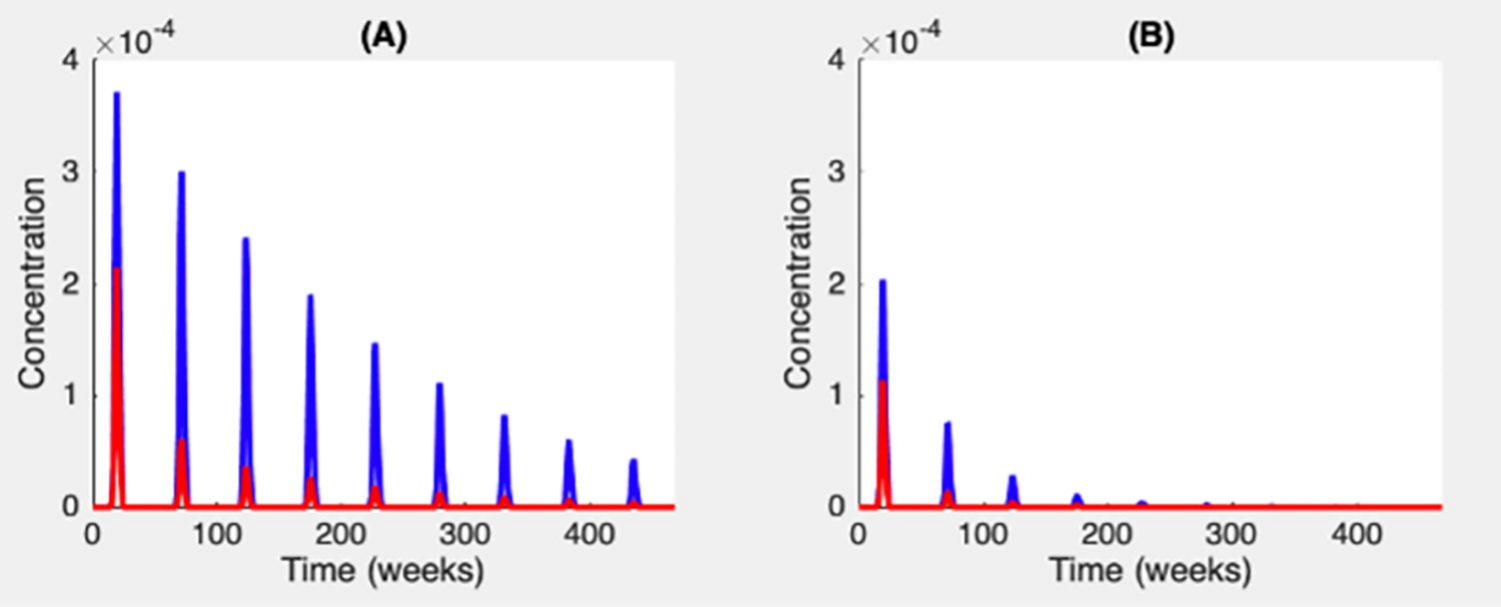
Secondary sources influences. Red curves represent the concentration of free-living bacteria provided by the primary source upstream to the lower catchment area. Blue curves represent the concentration of free-living bacteria provided by both primary and secondary sources to the lower catchment area. (A) saprophytic reproduction rate from dead fish ***σ***_F_ = 2 and saprophytic reproduction rate from dead macroinvertebrate ***σ***_A_ = 4. (B) ***σ***_F_ = 2 and ***σ***_A_ = 2.

The persistence of bacilli populations in river systems over time is mainly determined by two factors: the existence of sources upstream, and the successful attachment of bacilli to organic biomass, allowing bacterial reproduction on living and dead organisms. At *t* = 0, bacilli only exist in a single upstream primary source. Then, secondary sources are contaminated through biomass migration and water advection. As bacteria settle and reproduce in secondary sources, the sites located in alluvial plains received bacteria from both the primary and secondary sources. Fig 4 illustrates the respective importance, through time, of primary and secondary sources onto the population abundance dynamics of free-living bacteria in the alluvial plain. As long as bacterial sources continue to feed the overall catchment area every rainy season, this is enough to ensure at least an intermittent persistence of bacilli downstream. However, when this supply is interrupted, bacilli become more vulnerable to extinction in the alluvial plain. This can be explained by changing abiotic (e.g., temperature, *pH*) and biotic (e.g., fish and macroinvertebrate extinction) conditions, over the dry season, with demographic and environmental stochasticity of major importance in determining mycobacterial extinction risk as demonstrated by Garcia-Peña et al. [17]. In such a situation, higher bacilli production in secondary sources is mandatory for maintaining mycobacteria in the long term downstream. Indeed, after leaching, only bacteria attached to the organic biomass remain in the river. Therefore, biomass is essential for the persistence of bacilli throughout the year. Fig 4 illustrates the role of secondary sources over time. It is evident that secondary sources have a significant contribution over the years, depending on the local patch productivity in bacteria, namely the saprophytic and biomass-associated bacilli reproduction, and also on the biomass dispersion rate.

### 3.2. Biomass effects

**Fig 5:**
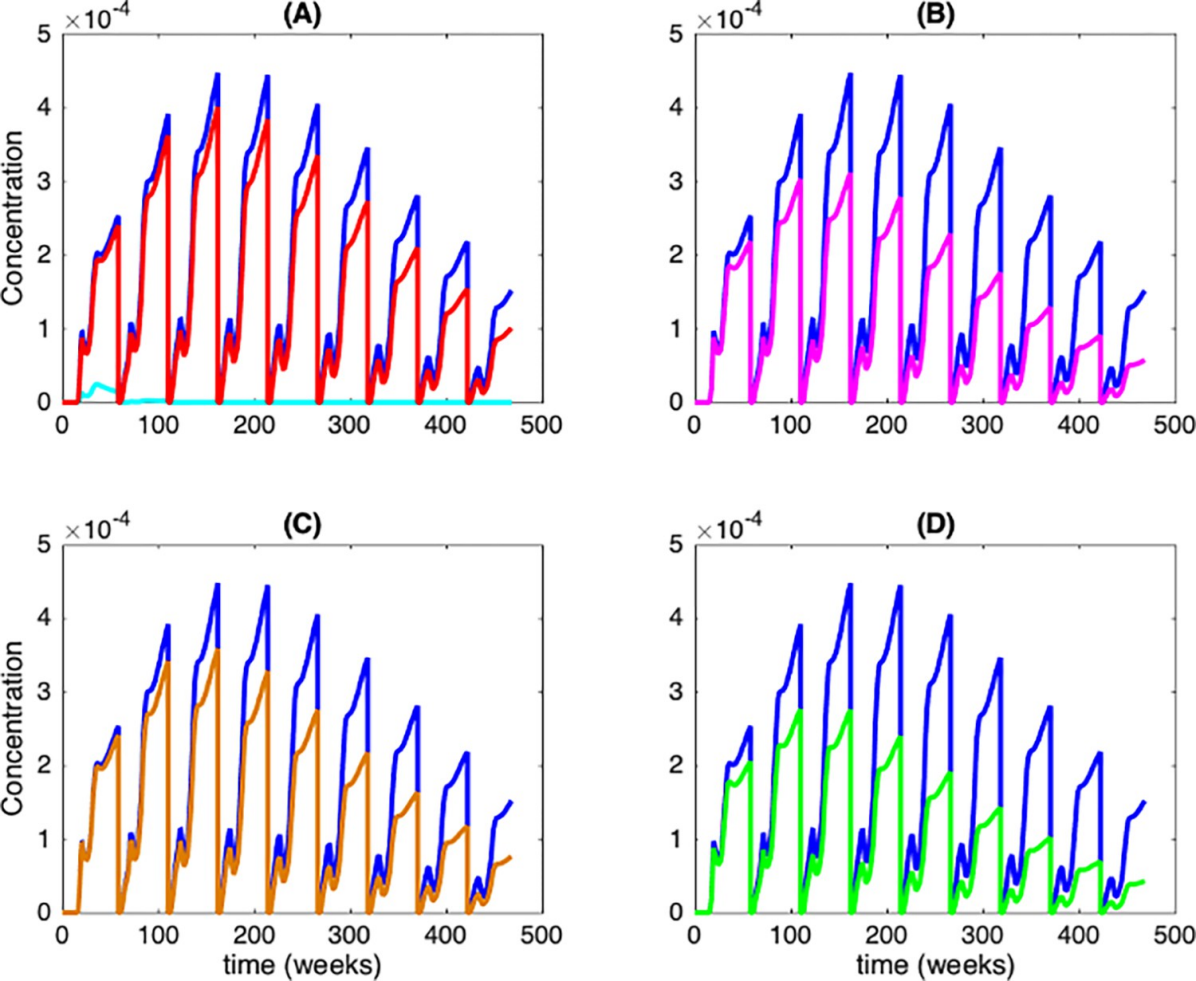
Relative sensitivity to biomass-related parameters. Panel (A) represents the relative contribution of fish and macroinvertebrate biomass types; the blue curve shows the dynamics when both biomass types are present, the red curve is that with absence of fish and that in cyan the dynamics observed in absence of macroinvertebrates. Parameters for illustration are: ***σ***_A_ = 2 and ***σ***_F_ = 4. Panel (B) illustrates the effect of reduction by 10% of the bacilli attachment rates on macroinvertebrate *ξ* (purple curve), panel (C) the effect of the same reduction by 10% on macroinvertebrate-associated bacilli growth rates ***θ*** (brown curve), and panel (D) the effect of the same reduction by 10% on bacilli release rate (saprophytism) from macroinvertebrate death ***σ***_A_ (green curve), on the abundance of the lower catchment area free-living bacteria. In each subfigure, the blue curve shows the dynamics given with initial values.

Fish-associated bacteria disperse at longer ranges than macroinvertebrate-associated bacteria, depending on the migration range of their respective host carriers. By contrast, higher bacterial attachment rates and higher reproduction on live and dead biomass are observed in nature for aquatic macroinvertebrates relative to the same events on fish. Fig 5 illustrates the influence of these two categories of organic biomass (i.e., freshwater fish and/or aquatic macroinvertebrates) (Fig 5A), bacilli attachment rate on macroinvertebrates (Fig 5B), macroinvertebrate-associated bacilli growth rate (Fig 5C), and bacilli release rate from dead macroinvertebrates (Fig 5D) on the abundance of free-living bacteria in alluvial plain.

Fig 5A illustrates the influence of the two categories of alive organic biomass (i.e., freshwater fish and aquatic macroinvertebrates) on abundance in free-living bacteria in lower catchment area, and clearly shows that aquatic macroinvertebrate biomass has a dominant effect in regulating the free-living bacilli abundance downstream. Basically, three main results are evident. When fish are absent (illustrated by a red curve in Fig 5A), we observe an average annual decline in bacilli presence in lower catchment area of only 16%. Conversely, in the absence of macroinvertebrates (illustrated by a cyan curve in Fig 5A), the bacilli dynamic downstream is strongly affected by an average annual decline of 97%. Since bacilli reproduction rate is higher on macroinvertebrate taxa than on fish, the macroinvertebrate biomass presence is logically the most important in the model. Table 2 summarizes statistics for this dominant mode of bacillus reproduction for various rates of saprophytic styles. In general, we can conclude from Table 2 that bacilli abundance in lower catchment area is largely explained by macroinvertebrate abundance upstream.

**Table 2:** Comparison of biomass influence on the average annual concentration of free-living bacilli in lower catchment area for different saprophytic rates. Percentage values give the reduction of bacilli abundance.

	Fish taxa absence	Macroinvertebrate taxa absence	All taxa
if ***σ***_A_ = ***σ***_F_	1.6037e-5 (-18.2%)	1.0640e-6 (-94.5%)	1.9607e-5
if ***σ***_A_ = 2***σ***_F_	13.966e-5 (-16.56%)	1.0640e-6 (-99%)	16.738e-5
if ***σ***_A_ = 3***σ***_F_	520e-5 (-3.7%)	1.0640e-6 (-99.9%)	600e-5

We then performed sensitivity analyses for three parameters describing the association between macroinvertebrates and mycobacterial bacilli: bacilli attachment rate on macroinvertebrate *ξ*, macroinvertebrate-associated bacteria growth rate ***θ***, and saprophytic reproduction rate from dead macroinvertebrate ***σ***_A_. For each scenario, we applied a 10% reduction to each of the three parameters in turn, where one was reduced while the two others remain constant. The impacts of such reductions on the abundance of free-living bacterial abundance in the alluvial plain are shown in Fig 5. Fig 5B shows the effect of a reduction by 10% of the bacilli attachment rate on macroinvertebrate *ξ*, Fig 5C the effect of a reduction by 10% of the macroinvertebrate-associated bacilli growth rate ***θ***, and Fig 5D the effect of a reduction by 10% of the saprophytic reproduction rate ***σ***_A_. It appeared that the mathematical model is very sensitive to a variation of the bacilli attachment rate ***ξ*** and of saprophytic reproduction rate ***σ***_A_, which have a similar impact on the bacilli density in this situation (Fig 5B and 5D). For example, the model predicts that a 10% reduction in macroinvertebrate-associated bacilli growth rate ***θ*** and saprophytic reproduction rate ***σ***_A_ will result into average annual reduction in bacterial downstream of approximately 26% and 44%, respectively (Fig 5C and 5D). However, we observe a cumulative negative effect of a reduction of bacterial growth rate on living macroinvertebrates (Fig 5C, red curve), which means that the model’s sensitivity to ***θ*** increases over time. This does not strongly affect the robustness of our mathematical model since we were more interested by intra- and medium-term inter-seasonal population dynamics of bacilli than by decadal or multi-decadal scenarios.

## 4 Discussion

In this paper, we provide a first mathematical model describing sapronotic pathogen dynamics in the context of aquatic ecosystems, considering two biomass-types as important components of the pathogen’s lifecycle. We used *Mycobacterium ulcerans* as an example of sapronotic agents based on the substantial medium- and long term ecological research done on this bacillus [33]. However, this first model can be easily adapted and generalized to other types of sapronoses. Understanding the spatial distribution and charge of these categories of sapronotic agents in ecosystems is a major challenge for understanding how animal and human epidemics occur among local populations, but also over larger spatial and global scales. However, major gaps exist because many of these microbial agents were initially unable to be characterized from the environment, and biomedical and veterinary research primarily focused on the clinical effects in animal vertebrates and humans. Given that many, if not all, sapronotic agents have an environmental reservoir, they share some similarities with host-parasite interactions that include a reservoir host [4]. Understanding such diseases nonetheless also implies understanding how the dynamics of sapronotic agents differ from those of host-parasite dynamics in the reservoir host.

For sapronotic diseases, we here introduce an important notion that the environmental reservoir is different from a reservoir host. Thus, we suggest using the term of environmental host carrier that can be alive or dead. Kuris et al. [4] considered a sapronotic agent as a free-living microorganism that obtains its nutrition by consuming organic matter derived from the decomposition of dead organisms. In the present model, we add to this assumption the possibility that such agents can also feed on living organisms. The mathematical model presented in this paper is sufficiently flexible to either keep these two possibilities of nutritional mode, or remove one of them. We kept the two options based on an important knowledge acquired on *M*. *ulcerans* in aquatic ecosystems. As empirically observed, it is then essential to understand the population dynamics of the different host carriers, which both serve to the sapronotic agent as substrates for nutrition and as dispersers in the environment. Given that free-living bacterial stages in the environment are finite in number, depending on seasonality and environmental stochasticity, their persistence and dispersal depend on host carrier density and biomass.

We have made the assumption that host carrier taxa (i.e., freshwater fish and macroinvertebrates) can disperse in all directions, with the fish able to spread sub-regionally or regionally over the river catchment area and macroinvertebrates only able to disseminate locally. We observed that when accepting larger dispersal rates for macroinvertebrates, this does not alter our main conclusions. Free-living sapronotic agents disperse with the main river flow (i.e., advection current), from upper source areas to downstream sink areas, where their effects are estimated by parameter values. We also assumed absence of ecological interactions between the two host carrier types and no bacterial transmission between them. This is important since it was considered that prey-predator relationship could be important in the transmission of *M*. *ulcerans* in aquatic ecosystems [34]. However, controversies still exist so that prey-predator relationships may have a minimal effect on bacilli dynamics [9], or indicate a supply-side effect of *M*. *ulcerans* efflorescence, i.e., a bloom-like phenomenon, in aquatic ecosystems [35]. Another mathematical modeling study is currently in progress for addressing the role of predation, i.e., active and passive, in the transmission of this type of sapronotic agent.

A main result from our study is that river catchment area topology (i.e., the existence of secondary sources) is important to consider for the transmission of this sapronotic disease agent. In fact, short coastal rivers are not sufficiently anastomosed to feed downstream areas in disease agent propagules, or their watersheds do not include upstream habitat sources favorable for the persistence of primary sources of such pathogens. Linear and simple catchment areas are also less favorable for the development of such sapronotic agents when compared to highly branched river systems of the same size. These assertions are supported by empirical evidence. Large watersheds or systems of smaller watersheds draining the same region are more likely to result in the accumulation of these sapronotic agents in alluvial plains where human populations are concentrated; e.g. Murray-Darling River in Australia [21], Nyong River in Cameroon, Central Africa [20], Benin and Ghana, western Africa [36], French Guiana, southern America [37,18]. This may be due to bacilli imports from particular soils and habitats in the headwaters of this wide watershed, which also has substantial agricultural development throughout its course [33]. Additional evidence is that of the Sinnamary watershed in French Guiana that has a hydroelectric dam: there have been drastically fewer cases of infections by *M*. *ulcerans* downstream after the dam’s construction [38]. Moreover, a similar situation of lower incidence associated with a large hydroelectric dam on the Volta River is found in the Volta region of Ghana [39]. This would tend to indicate that dams act as ramparts against downstream migration of bacteria, or that anoxic conditions of the lake were not favorable to the pathogen’s survival. Our spatially realistic metacommunity model captures the dynamics of natural river ecosystems, including seasonal variations, to simulate the accumulation of pathogenic mycobacteria in the lower reaches of a river basin. It therefore appears that hydrographic basins that are diverticulated and influenced by rainy periods inducing river mouth flooding, as in intertropical zones, capture source areas favorable to the maintenance and development of such sapronotic pathogens.

Another important finding is that taxonomic biomass load in the upper watershed area is important in explaining the mycobacterial abundance downstream, with macroinvertebrates being more important than freshwater fish by at least one order of magnitude. In addition, dispersal rates for both fish and macroinvertebrates are less important than upstream biomass for explaining mycobacterial load in lower catchment areas. This is essentially because such sapronotic agents may benefit from important biomass produced during the rainy periods, such as in fish and macroinvertebrates in intertropical belt. *M*. *ulcerans* may benefit from aquatic environments with elevated macroinvertebrate populations that provide increased chitinous compound availability (i.e., the main components of macroinvertebrate exoskeleton) and, thereby, enhance bacilli multiplication in such habitat patches through chitin conversion to glucosides [16]. On the contrary, fish scales are composed of both cosmin, which is a bone material, and keratin, an insoluble protein, which may not provide nutritive elements for bacilli; except for being a potential surface for attachment; and which would therefore better resemble a phoresy relationship. Furthermore, chitin is known for its antimicrobial properties against many *M*. *ulcerans* competitors, including bacteria, yeast and fungi [40]. The stimulating effects of chitin on *M*. *ulcerans* growth at low *pH*, i.e., under conditions that are not favorable for *M*. *ulcerans* in the absence of chitin, likely acts in synergy with chitin antagonistic effects on *M*. *ulcerans* competitors providing conditions of increased competitive ability and growth rates [41]. Irrespectively of the precise role played by chitin, i.e., being a simple material support or a nutrient source, significant effects observed on *M*. *ulcerans* growth relative to chitin concentration in an experimental study support a relationship between aquatic macroinvertebrates and spatio-temporal variation in occurrence and distribution of these mycobacteria in natural aquatic ecosystems [41]. Such a link observed in nature is strongly supported by the results of the present mathematical modeling study.

In ecological studies, evidence supports strong relationships between species richness and ecosystem functioning, specifically biomass production [42,43]. Our modeling results may also inform this biodiversity-disease relationship, i.e., species richness alteration due to human actions that have large impacts on biodiversity notably in removing large-bodied organisms from the ecosystems. This would tend to confirm an ecosystem-scale consequence of this phenomenon with reductions in large-bodied predator species (e.g., top predator fishes that prey upon micropredators and macroinvertebrates) leading to greater divergence between high- and low-biomass sites [38]. This finding has important implications for both the continued management of wetlands and river catchment areas, and their biodiversity, but also for better preventing emerging infectious sapronotic diseases that can develop on the destabilization in the organization of ecosystems.

In our modeling study, dispersal rates of both macroinvertebrates and fish are apparently much less important than biomass in explaining sapronotic agent charges in the alluvial plain. Fish are poorly infected, at least to our own knowledge from French Guiana where less than one fish per 200–250 specimens is reported infected by *M*. *ulcerans* [44]. Our mathematical model assumed that fish taxa were poorly surface-infected but can migrate long distances in catchment areas. In contrast, aquatic macroinvertebrates were considered highly infected on their body surface, but with relatively poorer dispersal capacity than fish. Overall, the model results indicate that in no scenario can taxon dispersion override the effect of biomass productivity for explaining the observed sapronotic agent charges downstream. This is one facet of the biodiversity-disease relationship that has never been discussed, i.e., natural biomass productivity in myriads of insects and other arthropods that have functional roles in ecosystems, and which contribute to the emergence and spread of animal and human sapronotic infections. Emerging disease agents, being zoonotic or sapronotic, are embedded within natural environments, with complex and important phenological interactions and population dynamics.

We observed from our mathematical modeling that the distribution of infected habitat patches is strongly influenced by the spatial dispersion capacity of host taxa. When hosts disperse very locally as for macroinvertebrates, the number of infected patches within the regional river catchment area varies slowly. In contrast, high dispersal of fish facilitates rapid spread of mycobacteria through the river, increasing thus the proportion of infected local habitat patches but not necessarily the mycobacterial load in the metacommunity. However, if there is strong heterogeneity in bacterial production among habitat patches, host carrier species migration can have either negative or positive effects on mycobacterial abundance. In particular, moving hosts from less productive to more productive patches increase bacterial growth. Furthermore, we found that mycobacterial abundance peaked when macroinvertebrates were present in all infected patches. Indeed, the absence of macroinvertebrates in patches reduces the local reproduction capacity of mycobacteria (see Fig 5A), thus explaining its concomitant depletion during the dry season in tropical areas. In the model, we assumed that host carrier births are local, but infectious free-living stages can enter or exit the system at a rate that is independent of the dynamics of host carriers. Of course, this infectious free-living stage growth depends not only on the biomass mortality rate but also on the differential reproduction rate of surface-associated- and saprophytic mycobacteria. Under such conditions, high levels of host-pathogen interactions can promote the persistence of high mycobacterial loads in river systems. Our model indicates that macroinvertebrate-associated mycobacteria are the more important contributors to the general life-cycle of these opportunistic pathogens, and that a saprophytic life-style and release of free-living propagules in water may also operate in concert for dead macroinvertebrates. Importantly, we emphasize that the effects of saprophytic processes are strongly dependent on biomass mortality rate, which was set at 5% in this study. This finding is supported by an experimental study on *Flavobacterium columnare*, a fish pathogen that is an increasing problem in freshwater fish farming [30]. This study demonstrated that the transmission of columnaris disease to living rainbow trout was most efficient from dead than living fishes due to the greater excretion of bacteria into the water from dead fish. These authors indicate that saprophytism may have been maintained as an effective transmission and survival strategy of *F*. *columnare*, with intense culture processes facilitating this transmission pathway. Unfortunately, transmission and survival in the environment of most, if not all, sapronotic disease agents is poorly known. Our modeling paper addresses both transmission and survival strategies, i.e., surface-associated residents and saprophytism, as plausible coexisting life-style strategies. Kuris et al. [4] propose a comprehensive list of possible candidates as sapronotic disease agents (see Box 2 in [4]), and our model could be applied to other animal or human pathogens such as *Brucella* spp., many other *Mycobacterium* and *Streptococcus* taxa, and even several fungal parasites, to name but a few.

A major limitation of our work is similar to that of most sapronotic diseases: the continuous and primary source of the agent is unknown. In other words, for such pathogens, it is often unknown whether the disease propagules repeatedly invade from outside the monitored systems or if the pathogen can persist in the environment outside contaminated hosts. Here, we used *M*. *ulcerans* as an environmental pathogen that is beginning to be better understood, but many parameter values (e.g., bacilli attachment rate on hosts, bacilli replication on living hosts and dead materials) still need to be potentially modified and improved based on empirical studies. Getting more precise information on these parameters could lead to improved modeling, thus we recommend new and innovative experimental studies to inform the parameters necessary to develop more efficient transmission models for sapronotic infections in animals and human. The question of the existence of ecological and evolutionary trade-offs is important to future work for understanding the relationships of virulence and ecosystem productivity, the persistence of dormant bacterial forms or the development of biofilms depending on abiotic and biotic conditions, or bacterial growth rate depending on different media, e.g. dead or alive biomass, and their quality either as simple substrate or as provider of nutritional resources. We invite and encourage microbiologists working on sapronotic pathogens to collaborate better with disease ecologists and mathematical modelers; and this to propose mathematical models of the transmission of sapronotic agents that are both as plausible as possible but also allow projections to be made on the future risks of emergence of this category of pathogenic agents.

## 5 Conclusion

Our study points to the crucial necessity of both understanding and modeling the transmission dynamics of sapronotic animal and human pathogens using an ecosystem-based approach. By uncovering the complex interplay between bacterial attachment, replication and environmental factors such as water flow dynamics in river catchments, our work provides valuable information on the spread and persistence of such pathogens in natural flowing systems. In addition, our results highlight the importance of secondary sources, i.e., the settlement of bacteria on new sites, in determining downstream bacterial abundance. This suggests the relevance of taking into account the spatial configuration and topology of river catchment areas in infectious disease management strategies.
